# Exhaled nitric oxide is not a biomarker for idiopathic pulmonary arterial hypertension or for treatment efficacy

**DOI:** 10.1186/s12890-019-0954-z

**Published:** 2019-10-29

**Authors:** Majid Malekmohammad, Gert Folkerts, Babak Sharif Kashani, Parisa Adimi Naghan, Zahra Habibi Dastenae, Batoutl Khoundabi, Johan Garssen, Esmaeil Mortaz, Ian M. Adcock

**Affiliations:** 1 Tracheal Disease Research Center, National Research Institute of Tuberculosis and Lung Diseases (NRITLD), Shahid Beheshti University of Medical Sciences, Tehran, Iran; 2 Division of Pharmacology, Utrecht Institute for Pharmaceutical Sciences, Faculty of Science, Utrecht University, Utrecht, Netherlands; 3 Chronic Respiratory Diseases Research Center, National Research Institute of Tuberculosis and Lung Diseases (NRITLD), Shahid Beheshti University of Medical Sciences, Tehran, Iran; 4 Helal-e-Iran Applied Science Higher Education Institute Red crescents society of Iran, Tehran, Iran; 5 Clinical Tuberculosis and Epidemiology Research Center, National Research Institute of Tuberculosis and Lung Diseases (NRITLD), Shahid Beheshti University of Medical Sciences, Tehran, Iran; 6 Cell and Molecular Biology Group, Airways Disease Section, Faculty of Medicine, National Heart and Lung Institute, Imperial College London, London, UK; 7 Priority Research Centre for Asthma and Respiratory Disease, Hunter Medical Research Institute, University of Newcastle, Newcastle, NSW Australia

**Keywords:** FeNO, Hypertention, Lungs, Exhaled nitric oxide

## Abstract

**Background:**

Idiopathic pulmonary arterial hypertension (IPAH) is a fatal illness. Despite many improvements in the treatment of these patients, there is no unique prognostic variable available to track these patients. The aim of this study was to evaluate the association between fractional exhaled nitric oxide (FeNO) levels, as a noninvasive biomarker, with disease severity and treatment outcome.

**Methods:**

Thirty-six patients (29 women and 7 men, mean age 38.4 ± 11.3 years) with IPAH referred to the outpatient’s clinic of Masih Daneshvari Hospital, Tehran, Iran, were enrolled into this pilot observational study. Echocardiography, six-minute walking test (6MWT), FeNO, brain natriuretic peptide (BNP) levels and the functional class of patients was assessed before patients started treatment. Assessments were repeated after three months. 30 healthy non-IPAH subjects were recruited as control subjects.

**Results:**

There was no significant difference in FeNO levels at baseline between patients with IPAH and subjects in the control group. There was also no significant increase in FeNO levels during the three months of treatment and levels did not correlate with other disease measures. In contrast, other markers of disease severity were correlated with treatment effect over the three months.

**Conclusion:**

FeNO levels are a poor non-invasive measure of IPAH severity and of treatment response in patients in this pilot study.

## Background

Pulmonary arterial hypertension (PAH), especially its idiopathic form (IPAH), is a rare and progressive disease, which increases lung vascular resistance, right heart failure, and eventually premature death [1–3]. The World Health Organization (WHO) has divided pulmonary hypertension (PH) into five groups based on the clinical and pathophysiological characteristics of patients. PAH is characterized by fibrosis, hypertrophy and network-like lesions [2]. IPAH, refers to PAH where there is no family history or known cause [1]. IPAH is very rare and has a poor prognosis with an average life expectancy of 2.8 years with survival probabilities of 68, 48 and 35% at one-year, 3 years and 5 years respectively [5]. The incidence of IPAH is estimated at about 1 per million and its prevalence is estimated to be 7 per million of the population in each year [2].

As a consequence of the non-specific symptoms, the diagnosis is usually delayed. The diagnosis is usually determined with echocardiography and right heart catheterization [4, 5]. Several clinical measures are used to follow the severity, progression and treatment outcome in these patients including echocardiography, hemodynamic parameters, six-minute walk test (6MWT) and biochemical markers such as troponin and natriuretic peptides [6–8]. Echocardiography is a noninvasive technique used for initial screening assessment of treatment outcomes because it provides valuable information on the hemodynamic status of the right heart such as pulmonary arterial pressure, size, and function of the heart cavity [9]. The WHO functional capacity (WHO-FC) test and the 6MWT are simple, repetitive and inexpensive tests and are recognized by the Food and Drug Administration and by the European Medical Agency for the investigation of the outcomes in PAH. Measuring exercise capacity in the early stages of PAH is advisable prior to the use of surrogates and as a measure of response to treatment [10–13].

Several biomarkers for the diagnosis and prognosis of PAH have been introduced including N-terminal pro-brain natriuretic peptide (NT-ProBNP) and troponin tests which correlate well with mortality [11, 14]. Despite this, there is still a requirement for a cheap and reliable relatively non-invasive marker that measures disease severity and progression and/or the response to treatment. Such a biomarker may significantly reduce the economic burden and help improve the outcome of PAH patients. The gaseous mediator nitric oxide (NO) is important in the pathogenesis of PAH as it is produced by epithelial and endothelial cells following enzymatic conversion of citrulline and arginine [15] and maintains low blood pressure in the pulmonary system [16–18]. Some studies have shown reduced expression of endothelial NO synthase (eNOS) in PAH and that eNOS mRNA expression correlates with exacerbations of PH. In contrast, other studies have reported increased eNOS expression in PAH patients and in animal models of PAH [18]. NO regulates the pulmonary circulation in response to hypoxia, vasodilatation and smooth muscle cell proliferation [19]. Fractional exhaled NO (FeNO) is routinely measured in many respiratory clinics [20] and some, but not all, studies indicate that FeNO levels in PAH patients are inversely related to disease severity and alter during treatment [20–22].

Considering the importance of PAH and the need for a noninvasive biomarker, we examined whether FeNO was a good biomarker of disease severity and response to treatment in a small cohort of IPAH patients in Iran.

## Materials and methods

This observational study was performed on 36 IPAH patients recruited from the pulmonary arterial hypertension clinic of the Masih Daneshvari Hospital, affiliated to Shahid Beheshti University of Medical Sciences, Tehran, Iran between 2015 and 2017. The sample size was powered according to previous studies measuring the difference in FeNO levels between PAH patients and healthy subjects. IPAH patients were selected from all the patients referred to the pulmonary arterial hypertension clinic from April 2015 to 2017 according to the standard hospital protocol and guidelines which required rejection of pulmonary embolism and diagnosis of heart disease as a cause of PAH. Subjects were invited to take part in the study after providing written informed consent under local ethics (IRSBMU.NRITLD.REC.1394.162).

Exclusion criteria included very sick patients, a history of asthma or seasonal allergies, history of respiratory infections in the last 6 weeks, age less than 16 years, current smoking and the presence of secondary causes of IPAH such as, heart embolism, lupus, HIV, and liver cirrhosis. Demographic information including age, sex, height and weight was recorded on admission as were the WHO-FC class, 6MWT (including distance traveled and oxygen saturation), pulmonary artery pressure during right ventricular catheterization and the Pro-BN*P* values at baseline and after 3 months of treatment.

FeNO was measured using a portable device (NO breath, Bedfont, UK) within the clinic. In brief, after deep inhalation for 2–3 s to achieve near to total lung capacity, the patient immediately exhaled into the NO analyzer with a constant flow rate (0.05 l per second) using a visual indicator as described previously [23]. FeNO was measured twice, with at least 30 s between tests, and the mean recorded. If there was > 10% variability the FeNO measurement was repeated.

For ethical reasons we did not alter the treatment regimens of the IPAH patients even if they had been on the same treatment for several years. Treatments included endothelin receptor antagonists (ERAs) such as bosentan, guanylate cyclase stimulants such as riociguat, phosphodiesterase type 5 (PDE5) inhibitors such as sildenafil and prostanoids such as epoprostenol, treprostinil and iloprost.

### Statistics

The data were analyzed using the statistical package IBM SPSS version 22.0 (Statistical Package for the Social Sciences, Chicago, IL). The categorical variables are expressed as proportions and frequencies. The continuous variables are summarized as means ± standard deviation. To explore the independent nature of categorical variables, the chi square test was used. *P* values < 0.05 were considered significant. Normal distribution of variables was assessed using the Kolmogorov-Smirnov test. The changes in echocardiographic parameters and the FeNO and NTpro-BNP variables during follow-up were analyzed using repeated measures ANOVA with Pearson correlation coefficients. Dunnett’s test was used as a post-test analysis following ANOVA assessment of between group data.

## Results

Baseline demographics of the patients are shown in Table 1. 29 female and 7 male subjects were recruited with a mean age of 38.4 ± 11.3 years. There was no significant difference in baseline FeNO between IPAH patients (5.5 ± 1.8 ppb) and healthy controls (8.1 ± 4.1 ppb) (Table 2). At the start of the study the mean duration of the disease was 3 ± 1 years with 12 patients (32%) with class III disease, 22 patients (60%) with class II disease and 3 patients (8%) with class I disease according to the New York Heart Association (NYHA) functional classification (Table 3) with baseline NT-ProBNP levels at 549 ± 562 pg/ml (Table 4). IPAH patients had a mean 6MWT of 373 ± 100 m at baseline (Table 5) and a mean pulmonary arterial pressure of 85.7 ± 18.5 mmHg (Table 6).

**Table 1:** Patient Demographics

Variable	Mean ± SD/n (%)
Gender (M/F)	7 (18%)/ 30 (81%)
Age (years)	38.4 ± 11.3
Height (cms)	163.0 ± 8.0
Weight (Kg)	64.0 ± 9.4
BMI (Kg/m^2^)	23.9 ± 3.0
Disease Duration (years)	3.0 ± 1.0
NYHA functional classification	
Class I	3 (8%)
Class II	22 (60%)
Class III	12 (32%)
NT-ProBNP at baseline (pg/ml)	549 ± 562
mPAP (mmHg)	57.0 ± 12.0
Systolic PAP (mmHg)	89 ± 16
Diastolic PAP (mmHg)	37 ± 12

*NYHA* New York Heart Association, *mPAP* mean pulmonary arterial pressure

**Table 2:** Fractional nitric oxide (FeNO) measurements over time

Time	Mean (ppb)	Max	Min	SD	*P* value
Start of study	5.5	9	2	1.8	
After 3 months	6.5	16	2	2.3	0.272
Healthy subjects	8.1	20	5	4.1	0.632

**Table 3:** WHO functional class changes after 3 months therapy

Time	WHO Class			*P*-value
	I	II	III	
Start of Study	3 (8%)	22 (61%)	11 (30%)	
After 3 months	5 (14%)	18 (48%)	14 (38%)	0.539

**Table 4:** Changes in NT-ProBNP (pg/ml) levels in patients following the treatment

Time	Mean (pg/ml)	Max	Min	SD	*P* Value
Start of study	549	2450	62	562	
After 3 months	462	2085	70	484	0.030

**Table 5:** Distance travelled in metres (m) in the 6MWD test of patients after treatment

Time	Mean (m)	Max	Min	SD	*P* value
Start of study	373	543	150	100	
After 3 months	383	550	174	99	0.328

**Table 6:** Changes in pulmonary arterial pressure (mmHg) in patients undergoing echocardiography

Time	Mean (mmHg)	Max	Min	SD	*P* value
Start of study	85.7	115	55	18.5	
After 3 months	78.7	120	50	20	0.011

Continuing patients’ therapy for 3 months had no effect on FeNO levels (5.5 ± 1.8 versus 6.5 ± 2.3 ppb, *P* = 0.272) (Table 2) nor on the percentages of patients in each WHO functional class (Table 3, *P* = 0.539) compared to baseline. In addition, there was no difference in the mean 6MWT distance after 3 months extended therapy (Table 5, *P* = 0.328). In contrast, BNP levels before and after 3 months follow-up were significant decreased (549 ± 562 versus 462 ± 484 pg/ml, *P* = 0.03) (Table 4) as was mean pulmonary arterial pressure measured by echocardiography (PAPecho) (85.7 ± 18.5 versus 78.7 ± 20 mmHg, *P* = 0.011) (Table 6).

In contrast to the group mean data, there were significant differences in these parameters in patients across the WHO-FC groups although these did not vary significantly with treatment (Table 7).

**Table 7:** Comparison expiratory NO and hemodynamic variables in the three WHO functional classes before and after treatment

Time	Items	I		II		III		*P* value
before		Mean	SD	Mean	SD	Mean	SD	
	NT-ProBNP	170	96	324	209	963	849	0.003*
	6MWD	409	66	433	78	285	68	0.001*
	PAPS	68	7	82	15	95	20	0.030*
	FeNO	9	3	5	2	9	6	0.050*
	MPAP	55	7	53	12	58	14	0.311
after	NT-ProBNP (pg/ml)	197	88	353	155	895	749	0.001*
	6MWD	441	72	426	69	306	70	0.001*
	PAPS	66	40	71	13	92	21	0.040*
	FeNO	7	2	6	2	8	6	0.478
	MPAP	48	13	56	10	56	12	0.659

**Fig. 1:**
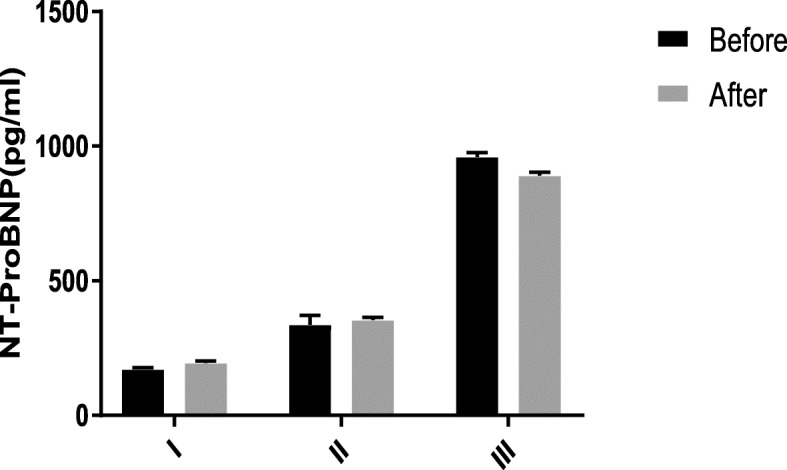
N-terminal pro b-type natriuretic peptide (NT-ProBNP) changes in the three WHO functional classes before and after treatment. Levels of NT-ProBNP before and after treatment in class I (*n* = 8 subjects), II (*n* = 17 subjects) and III (*n* = 11 subjects). Data are reported as mean ± sem

**Fig. 2:**
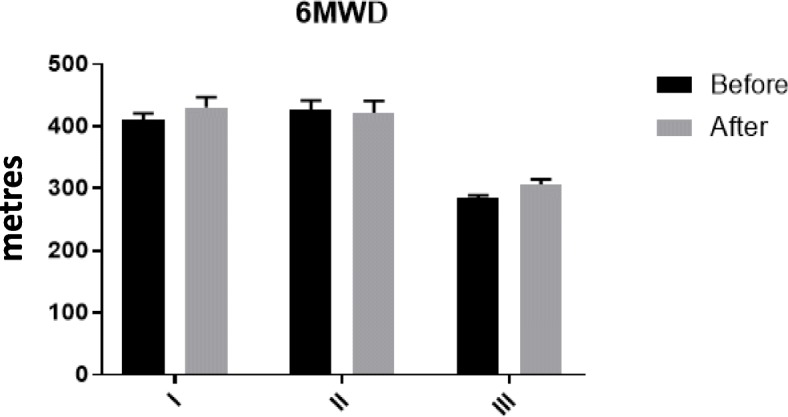
Six minute walk distance (6MWD) changes in the three WHO functional classes before and after treatment. The effects of treatment on the 6MWD in class I (n = 8 subjects), II (n = 17 subjects) and III (n = 11 subjects). Data are presented as mean ± sem

The decrease in NTproBMP levels had a significant and strong correlation with WHO-FC before (r = 0.7, *p* = 0.001) and after treatment (r = 0.6, *P* = 0.001) (Table 7 and Fig. 1). In addition, there was an inverse correlation between the level of NT-ProBNP and the 6MWT (r = − 0.67, P = 0.001) at baseline and after 3 months of observation (Fig. 2). The level of NT-ProBNP was also significantly correlated with PAP in angiography (r = 0.78, P = 0.011) and negatively correlated with the 6MWD (r = − 0.8, P = 0.001). This indicates that higher NT-ProBNP levels associate with lower distances covered in the 6MWD test and with higher WHO-FC.

## Discussion

This study was conducted to examine whether FeNO levels had any association with IPAH disease presence or outcomes following 3 months continued therapy with the same treatment patients were on at baseline. Although the mean level of FeNO in IPAH patients at baseline was lower than in healthy subjects, this was not statistically significant, nor did FeNO significantly alter over the 3 months study duration. FeNO was not correlated with 6MWD, NT-proBNP, WHO-FC class, PAPecho and mean pulmonary arterial pressure measured with angiography, either before or after treatment.

In the current study we demonstrated that NT-proBNP, 6MWD, WHO-FC, systolic PAPecho measures were significantly correlated and increases in NT-proBNP, PAPecho and WHO-FC were inversely correlated with the distance walked in the 6MWT. The correlation did not change with 3 months continued therapy possibly reflecting either too short a duration of treatment or the fact that patients were maintained on the same treatment and any changes that could potentially result from treatment may already have occurred. Thus, potential changes that would otherwise have occurred with the onset of changes in treatment may not have been seen in these patients.

Previous studies examining the levels of FENO or exhaled NO in PH have reported increased, decreased and similar levels compared to healthy non-PAH controls. This may reflect the different types of PH investigated in class 1 PH (PAH) since PAH may be classified into subgroups of IPAH such as PAH caused by certain drugs, PAH associated with connective tissue diseases or with congenital heart disease [24, 25].

Small studies in IPAH patients including that of Kaneko on 8 patients with IPAH [26], the Girgis study on 10 patients with PAH (8 patients with IPAH and 2 patients with PAH related to medicine) [22] and the Ozkan study with slightly larger studies on 22 patients [27] all indicated that FeNO levels were lower in PAH patients compared with healthy subjects.

In contrast, FeNO levels were similar in healthy subjects and in patients with IPAH at rest [28] and in patients with PAH associated with scleroderma [29]. FeNO levels did not rise in patients with IPAH after exercise whereas they increased in healthy subjects following exercise [28]. These results are similar to our data as all subjects were studied at rest despite the fact that they examined ILD-related PAH patients [28]. Furthermore, 21 patients with scleroderma-associated PAH had similar FeNO levels to healthy subjects [29]. Finally, at least two studies in drug- and heart disease-associated PH demonstrated enhanced FeNO levels compared to healthy controls [30, 31]. These differences may be due, at least in part, to differences in flow rates used for the detection of FeNO which highlights the need to use standardized flow rates [32]. Other factors such as consuming nitrate-rich foods, drinking water and caffeine and alcohol consumption can also affect FeNO levels and should be measured as confounders [33, 34].

Although we report no statistically significant decrease in FeNO measurements in our IPAH patients versus controls, there was a small difference in the mean levels (5.5 vs 8.1). Smaller studies may have reported an erroneous significant decrease in FeNO as a result of the number of subjects in each group. It is possible that a much larger study group would show significance but the difference in mean FeNO levels is small and its usefulness as a biomarker would still be questionable at the individual subject level. In addition, other factors that affect FeNO measurements including exercise, current smoking and viral infections of the upper and lower respiratory tract [35] should be treated as confounders in all future studies.

NT-proBNP is a widely used PH biomarker that has been shown to have prognostic value in several studies and can indicate a response to treatment [35]. In this study, although other parameters did not change, NT-proBNP was significantly decreased over 3 months. This correlated with changes in a number of clinical variables such as PAPecho, 6MWD and WHO-FC. Although BNP is a relatively good biomarker, there is still a need for an ideal biomarker with a high sensitivity and specificity for different types of IPAH, easy and rapid measurement in breath or in a simple blood assay and, ideally, with little or no overlap in values between health and disease. In addition, there is a need for a biomarker of potential treatment response to distinct therapies including prostanoid therapy, combination therapy and/or non-group 1 patients who are likely to benefit from off-label use of oral therapy.

There are several strengths to this study. This is the first investigation of the use of FeNO as a biomarker for PAH in Iran and we also studied more IPAH patients than in most previous studies using standardized flow rates. In contrast, most previous studies were conducted in patients with PAH due to other causes and were not restricted to IPAH. However, many factors can affect FeNO measures and these need to be considered in all future studies which makes generalization difficult and may affect the comparison of the results.

FeNO measurements may potentially vary according to the device(s) used in the cited PAH/FeNO literature. The NObreath® device is similar to other devices used in the field with a concentration range of 0-500 ppb and a repeatability of, and accuracy of, ±5 ppb for values ≤50 ppb and can be used between 15 and 35 °C [36]. This device is recommended by the UK National Institute for Clinical Excellence (NICE) and conforms to American Thoracic Society (ATS) and European Respiratory Society (ERS) guidelines [37]. The major difference between the NObreath® device and other devices such as the NIOX-MINO® device is that these other devices will only provide data at a given exhaled flow rate. In contrast, NObreath accepts samples produced using poor exhalation maneuvers but this does, however, allow detection of FENO in subjects such as children and those with severe disease where flow rate compliance is sub-optimal [38].

## Conclusion

This study reports no differences in FeNO between healthy controls and IPAH patients. In addition, continued treatment for 3 months had no significant effect on FeNO levels in IPAH patients. FeNO did not significantly correlate with other biomarker or clinical variables such as NT-proBNP, 6MWD, WHO-FC or PAPecho. Despite the limitations of the study around low patient numbers, short duration of follow-up, the use of multiple treatment regimens and no switch of treatment, the data do not support the use of FeNO in motoring IPAH. Further larger studies of a longer duration are needed to confirm these results.

## Data Availability

All data generated or analyzed during this study are included in this published article and its supplementary information files.

## Abbreviations

6MWT: Six-minute walk test
BNP: Brain natriuretic peptide
IPAH: Idiopathic pulmonary arterial hypertension
NO: Nitric oxide
NT-ProBNP: N-terminal pro-brain natriuretic peptide
PAH: Pulmonary arterial hypertension
