# Correction to: Exhaled nitric oxide is not a biomarker for idiopathic pulmonary arterial hypertension or for treatment efficacy

**DOI:** 10.1186/s12890-019-0984-6

**Published:** 2019-11-05

**Authors:** Majid Malekmohammad, Gert Folkerts, Babak Sharif Kashani, Parisa Adimi Naghan, Zahra Habibi Dastenae, Batoul Khoundabi, Johan Garssen, Esmaeil Mortaz, Ian M. Adcock

**Affiliations:** 1 Tracheal Disease Research Center, National Research Institute of Tuberculosis and Lung Diseases (NRITLD), Shahid Beheshti University of Medical Sciences, Tehran, Iran; 2 Division of Pharmacology, Utrecht Institute for Pharmaceutical Sciences, Faculty of Science, Utrecht University, Utrecht, Netherlands; 3 Chronic Respiratory Diseases Research Center, National Research Institute of Tuberculosis and Lung Diseases (NRITLD), Shahid Beheshti University of Medical Sciences, Tehran, Iran; 4 Helal-e-Iran Applied Science Higher Education Institute Red crescents society of Iran, Tehran, Iran; 5 Clinical Tuberculosis and Epidemiology Research Center, National Research Institute of Tuberculosis and Lung Diseases (NRITLD), Shahid Beheshti University of Medical Sciences, Tehran, Iran; 6 Cell and Molecular Biology Group, Airways Disease Section, Faculty of Medicine, National Heart and Lung Institute, Imperial College London, London, UK; 7 Priority Research Centre for Asthma and Respiratory Disease, Hunter Medical Research Institute, University of Newcastle, Newcastle, NSW Australia

**Correction to: BMC Pulm Med**

**http://dx.doi.org/10.1186/s12890-019-0954-z**

Following publication of the original article [1], the authors flagged that the name of the author ‘Batoul Khoundabi’ had been provided with an incorrect spelling: ‘Batoutl’ was given in place of ‘Batoul’.

This typo has now been corrected in the original article and the corrected version is provided with this correction.
